# Identifying Unique Neighborhood Characteristics to Guide Health Planning for Stroke and Heart Attack: Fuzzy Cluster and Discriminant Analyses Approaches

**DOI:** 10.1371/journal.pone.0022693

**Published:** 2011-07-28

**Authors:** Ashley Pedigo, William Seaver, Agricola Odoi

**Affiliations:** 1 Department of Comparative Medicine, The University of Tennessee, Knoxville, Tennessee, United States of America; 2 Department of Statistics, Operations and Business Science, The Universtiy of Tennessee, Knoxville, Tennessee, United States of America; Innsbruck Medical University, Austria

## Abstract

**Background:**

Socioeconomic, demographic, and geographic factors are known determinants of stroke and myocardial infarction (MI) risk. Clustering of these factors in neighborhoods needs to be taken into consideration during planning, prioritization and implementation of health programs intended to reduce disparities. Given the complex and multidimensional nature of these factors, multivariate methods are needed to identify neighborhood clusters of these determinants so as to better understand the unique neighborhood profiles. This information is critical for evidence-based health planning and service provision. Therefore, this study used a robust multivariate approach to classify neighborhoods and identify their socio-demographic characteristics so as to provide information for evidence-based neighborhood health planning for stroke and MI.

**Methods and Findings:**

The study was performed in East Tennessee Appalachia, an area with one of the highest stroke and MI risks in USA. Robust principal component analysis was performed on neighborhood (census tract) socioeconomic and demographic characteristics, obtained from the US Census, to reduce the dimensionality and influence of outliers in the data. Fuzzy cluster analysis was used to classify neighborhoods into Peer Neighborhoods (PNs) based on their socioeconomic and demographic characteristics. Nearest neighbor discriminant analysis and decision trees were used to validate PNs and determine the characteristics important for discrimination. Stroke and MI mortality risks were compared across PNs. Four distinct PNs were identified and their unique characteristics and potential health needs described. The highest risk of stroke and MI mortality tended to occur in less affluent PNs located in urban areas, while the suburban most affluent PNs had the lowest risk.

**Conclusions:**

Implementation of this multivariate strategy provides health planners useful information to better understand and effectively plan for the unique neighborhood health needs and is important in guiding resource allocation, service provision, and policy decisions to address neighborhood health disparities and improve population health.

## Introduction

Stroke is the third most common cause of death and leading cause of debilitation in the US [1]. Coronary heart disease, including myocardial infarction (MI), accounts for nearly 1 out of every 6 deaths in the US [2]. These health conditions are serious burdens to the US health system with prevalence estimates of 2.9% and 3.6% and annual costs estimated at $73.7 and $177.1 billion for stroke and MI, respectively [2].

These burdens vary by demographic, socioeconomic, and geographic factors. Several studies have reported geographic variations in prevalence and mortality of stroke and MI with the highest risks being reported in southeastern US [1], [3], [4] and in populations living in rural areas [3]–[5]. Tennessee ranks 3^rd^ and 4^th^ highest in the US for stroke and coronary heart disease including MI, respectively [2]. The 2006 annual age standardized mortality risks of stroke and MI in Tennessee were 67.5 and 85.5 deaths per 100,000 persons, compared to the national risks of 53.5 and 58.9 deaths per 100,000 persons, respectively [6]. Many rural areas of Tennessee, including the Appalachian Region, form part of the “stroke belt” of the US [3], [4], [7]. Populations that are male [1], [3], [8], [9], black [3], [7], [10], or 60–65 years of age and older [1], [3], [11], [12] have higher stroke or MI prevalence and mortality than other demographic groups. The relationships with socioeconomic factors are predominantly described as inverse with increasing risk of stroke or MI being associated with decreasing levels of income [8], [11], [13], [14], education [1], [11], [15], and composite measures of socioeconomic status (SES) or deprivation that include factors like employment, occupation, single parenthood, marital status, housing value or housing ownership, to mention but a few [8], [13], [16], [17].

Although socioeconomic, demographic, and geographic factors are known to be important determinants of stroke and MI, little is known regarding the clustering of these risk factors in neighborhoods. Research has overwhelmingly found that an individual's health can be influenced by the socioeconomic and demographic characteristics of their neighborhood beyond their individual characteristics [11], [18]–[20]. Clustering of these determinants of health across neighborhoods inevitably impacts health outcomes and thus health planning. Therefore, research should focus on identifying disparities among sub-groups to better understand health needs at the neighborhood level and guide health programs geared toward reducing/eliminating these disparities [7], [18]. Moreover, the multi-factorial nature of disease determinants implies that as many risk factors as reasonably possible need to be included for the most realistic analyses. Thus, the analysis of the complex and multidimensional nature of socioeconomic, demographic, and geographic risk factors requires the use of multivariate approaches [7], [11], [18], [21], [22].

With these issues in mind, the objective of this study was to classify neighborhoods in East Tennessee (using multivariate techniques) based on demographic, socioeconomic, and geographic risk factors for stroke and MI to better identify and understand population characteristics and health needs at the neighborhood level to support population health planning and policy. Many of these risk factors are expected to be interdependent, such that clusters based on these characteristics will not be mutually exclusive. Thus, this study uses multivariate methods (robust principal components analysis, fuzzy cluster analysis, discriminant analysis, and classification trees) to address this issue.

## Materials and Methods

### Ethics Statement

This study was approved by the University of Tennessee Institutional Review Board (IRB #: 7584B).

### Study area population

This study was performed in the East Tennessee Appalachian region, an area that includes eleven counties: Claiborne, Cocke, Grainger, Greene, Hamblen, Hancock, Hawkins, Jefferson, Knox, Sevier, and Union. These counties were chosen because of their high risk of stroke and/or MI. This area has a population of just over 857,000 and includes 168 census tracts (CTs). Census tracts are statistical subdivisions of a county that have between 2,500 and 8,000 persons, do not cross county boundaries, and are homogenous with respect to population characteristics, economic status, and living conditions [23]. The US Census Bureau further describes the design of CTs to provide a relatively stable set of geographic units that allow statistical comparisons of population characteristics between decennial censuses. Additional information on how the boundaries of the CTs are determined can be found at the US Census Bureau [24]. Census tracts have been shown to be good proxies of natural neighborhood boundaries and are thus useful in describing neighborhood population characteristics, as well as health needs [21], [25]. Furthermore, other studies of socioeconomic characteristics in the US have used census tracts to represent neighborhoods [26], [27]. Given these characteristics, CTs were used in this study to represent neighborhoods as the geographical unit of analysis and therefore all analyses, results, and inferences were made at this population level.

### Data acquisition

#### Population characteristics

Census tract level socioeconomic, demographic, and population data for the study area were obtained from the census 2000 summary file 3, [28]. Since these data are available in the US only through the decennial census, the 2000 data was deemed best suited to match the disease data (1999–2007). The variables considered in the study were those that have been reported in the literature [8], [13], [16], [17] to be associated with risk of stroke and MI either independently or as part of a composite measure. They include: race, gender, age (40–49, 50–59, 60–64, 65 years and older), marital status (for population 15 years and older), population living below poverty, per capita income, educational attainment (less than high school, high school graduate, some college, bachelor degree, or graduate degree), single parent households, housing ownership, housing value, and the urban/rural classification of each neighborhood.

#### Mortality data

Mortality data covering the period 1999–2007 were obtained from the Tennessee Department of Health and were used for comparison of mortality risks across neighborhoods. Stroke and MI mortality cases were defined using ICD 10 codes I60–I69 and I21–I22, respectively. Mortality case addresses were geo-coded using Batch Geocode [29] and imported into ArcGIS 9.3 [30]. Point-in-polygon join was used to connect the mortality data to the census tract cartographic boundary files obtained from the U.S. Census Bureau [31].

### Data analysis

#### Data management

With the exception of income and housing value, all variables were analyzed as the proportion of the population in each CT (neighborhood). One neighborhood in Knox county, that had a population of 232 and included a mental health facility, was removed from the analysis due to missing data values for many variables.

#### Robust principal components analysis (PCA)

When the ultimate goal of the analysis is to identify group structure within data using cluster analysis based on many variables, principal components analysis (PCA) can be used to reduce the dimensionality of the data [32]. This process reduces bias in clustering since substantial interdependencies, or high correlations, often exist among the many variables being considered. However, outliers can also bias the orthogonal linear combinations, as well as the cluster formation. Thus in this study, robust PCA in NCSS [33] was performed to reduce the dimensionality of the 22 strongly interdependent socioeconomic and demographic variables and to decrease the influence of outliers prior to subsequent cluster analysis [34], [35]. This method uses weights that are inversely proportional to the degree to which an observation is outlying [36]. The robust PCA was performed on the correlation matrix, which has values standardized by variance for the whole dataset, instead of just one variable, since major differences in variability and scale were expected amongst these variables [37]. Kaiser's eigenvalue cutoff of 1.0 was used to retain five components that accounted for 80% of the variation [38]. The five retained component scores (with a mean of zero and variance of 1.0) were multiplied by the square root of their eigenvalues to retain maximum-ordered variances. This was done to ensure that principal components with high variances would have more weight in subsequent cluster analysis.

#### Fuzzy cluster analysis

Clustering techniques can be used on the robust PCA scores to find groups or clusters that contain observations with similar socioeconomic and demographic characteristics [35]. Typically, there is a hard or crisp assignment of observations into clusters, such as with k-means. However, a generalization of the k-means clustering algorithm (called fuzzy k-means clustering) allows observations to have a non-crisp assignment to clusters [39]. This non-crisp assignment allows observations to have a degree of belonging to two or more clusters [35], i.e., some observations may partly belong to other clusters.

The fuzzy K-means clustering algorithm is based on minimizing the following objective function:
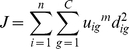
(1)where u_ig_ is the degree of belonging of the i^th^ observation to the g^th^ cluster [35], [39], m is the fuzzifier (m≥1: m = 1 or close to 1 gives a crisp solution; and as m increases greater than 1, the solution becomes more and more fuzzy with each increment); and d^2^_ig_ is a Euclidean measure of distance based on the robust principal component scores. With the computation of the degrees of belonging, there is a re-estimate of the cluster centroids in a fuzzy way according to the following relationship:
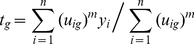
(2)In this case, i = 1, 2, …, n observations, g = 1, 2, …, r clusters, and y_i_ is the robust principal component score in this study. There is an iterative computation of Euclidean distances relative to the cluster centroids. New values of u_ig_, which minimize J (equation (1)) for given distance measures, can be computed by:
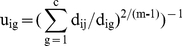
(3)where i = 1, 2, …, n observations, j = 1, 2, …, n observations, g = 1, 2, …, r clusters. The minimization of equation (1) with respect to the centroids (equation 2) and the degree of belonging (equation 3) continues until the differences between successive membership matrices are less than some pre-assigned value (in this study the value is 0.001).

The fuzzy clustering strategy allows a sensitivity analysis on cluster structure as well as assessment of the uniqueness of each observation to a particular cluster by varying the fuzzifier and the number of clusters. The fuzzifier is increased typically by small amounts from 0.10 up to 0.25. Some data sets will be extremely sensitive to changes in the fuzzifier and others not [40]. The tremendous amount of information provided by the degree of belonging information can be summarized using either (a) Dunn's normalized partition coefficient (FPU), with values closer to one reflecting hard partition and values closer to zero fuzzy solutions; or (b) the normalized average squared error (DPU), where values closer to zero indicate hard solutions and values near one are fuzzy solutions [39], [41], [42]. The solution that will provide the best insight to the cluster structure of the data, in this case the population profiles of neighborhoods (observations), should be neither too hard nor too fuzzy [35]. This is addressed with the fuzzy indices, FPU and DPU, and with the validation of classification into each cluster with discriminant analysis with the original variables. A more comprehensive discussion on the selection of a fuzzy solution (i.e. number of clusters and fuzzifier), is available in Seaver, et al (2004) [43].

In this study, fuzzy cluster analysis was performed in NCSS [33] using the principal component scores from robust PCA of the population characteristics to identify peer neighborhoods (PNs). In order to identify the solution with the most distinction between PNs, a sensitivity analysis was performed by varying the fuzzifier from 1.0 to 1.6 and the number of clusters from 3 to 6, based on the suspected group structure of the study area.

#### Validation of PNs

After identifying PNs, it was important to assess accuracy of PN identity, identify misclassified neighborhoods, and determine the characteristics most important for separating the neighborhoods. This was done using: (a) non-parametric nearest neighbor discriminant analysis (DA) with two neighbors (k = 2) in SAS 9.2 [44] and (b) classification and regression tree (CART) in AnswerTree 3.0 [45]. The performance of the DA was evaluated by estimating error rates (or probabilities of misclassification) in the classification of neighborhoods using cross validation (or jack-knife) method where *n−1* neighborhoods were used to predict the classification of the neighborhood held out [46].

The means of socioeconomic and demographic variables were compared in each PN between misclassified and non-misclassified neighborhoods using Hotelling's two sample t-test to investigate characteristics of the misclassified neighborhoods. Randomization tests of significance were used since the assumption of multivariate normality was not met [36], [47].

When distributional assumptions are uncertain and more flexibility is needed, classification (decision) trees can be used to predict the assignment of observations into discrete groups based on one or more predictor variables. One particular advantage of classification trees is that they readily lend themselves to being displayed graphically, making them easier to interpret and use. Classification trees construct hierarchical decision rules in the form of binary trees starting with the original classification for the data and ending with somewhat homogeneous groups of observations. Computationally, decisions must be made on: the criteria for predictive accuracy, selecting splits, stopping point for splitting, and selecting the “right-sized” tree. However, the goal in this study was simplicity of the tree and ease in comparison with the traditional nearest neighbor results to validate the uniqueness of identified PNs. Thus, CART [45] with binary splits at four levels was used.

#### Comparison of mortality between peer neighborhoods (PN)

Annual age-adjusted mortality risks of PNs for stroke and MI were calculated by the direct standardization method in Stata 10 [48] using the 2000 Tennessee population as the standard population. A two sample test of equality of proportions for each PN pair was performed and the p-values adjusted for multiple testing using the Simes method [49]. Spatial distribution of identified PNs were displayed in ArcGIS 9.3 [30].

## Results

### Robust principal components analysis

The five retained components from robust PCA explained 80% of the variation in the data. The first component explained 34% of the variation and was primarily composed of socioeconomic (education, income, housing value, employment) and geography (urban versus rural) variables (Table 1). Demographic variables (race, single parent families, married population, and home ownership) were heavily loaded onto component 2, which explained the next largest portion (26%) of the variation. Component 3 was also a demographic perspective of the data, with average family size and age primarily loaded on this component. Variables for race and gender were also important for component 3. Components 4 and 5 have less clear interpretations. Component rotation, using Varimax rotation (results not shown), did not change the loadings or interpretation, except to make a few variables more distinct for components 4 (race and age) and 5 (gender and rural geography). A regular PCA on the current data (results not shown) yielded the same percentage of total variation explained (80%); however, the first three components explained less variation individually compared to the robust (Table 1). By adjusting for outliers in the robust PCA, the variation is more distinctly partitioned in the components, allowing for better interpretation.

**Table 1:** Component Loadings from Robust Principal Components Analysis for Socioeconomic and Demographic Variables.

	Components				
Variables	1	2	3	4	5
% of variation explained	34%	26%	9%	6%	5%
Living in urban area	−0.69	0.57	0.09	−0.03	0.17
Living in rural area	0.56	−0.40	−0.04	−0.04	−0.36
White race	0.09	−0.79	0.34	0.32	0.12
Black race	−0.02	0.74	−0.35	−0.37	−0.10
Male	0.22	−0.55	−0.15	0.33	−0.50
Age 40–49 years	−0.29	−0.44	−0.48	−0.38	0.05
Age 50–59 years	0.12	−0.68	0.02	−0.34	−0.05
Age 60–65 years	0.39	−0.47	0.27	−0.39	−0.01
Age over 65 years	−0.04	0.27	0.73	−0.49	0.15
Single parent families	0.27	0.73	−0.35	−0.04	0.16
Average family size	0.09	0.00	−0.84	−0.06	0.20
Married	0.03	−0.90	−0.18	−0.10	0.16
Employed	−0.70	−0.34	−0.15	0.36	0.22
Per capita income	−0.88	−0.27	0.01	−0.19	−0.09
Homeowners	0.10	−0.88	−0.19	−0.19	0.15
Less than high school degree	0.92	0.03	−0.01	−0.09	−0.07
High school degree	0.86	−0.18	0.04	0.04	0.27
Some college education	−0.79	0.17	0.11	0.29	0.08
Bachelor degree	−0.94	0.01	−0.02	−0.05	−0.18
Graduate degree	−0.86	−0.04	−0.01	−0.22	−0.32
Below poverty	0.63	0.60	−0.05	0.04	−0.35
Median housing value	−0.83	−0.31	−0.04	−0.10	−0.13

### Identified peer neighborhoods

#### Fuzzy cluster analysis results

**Figure 1:**
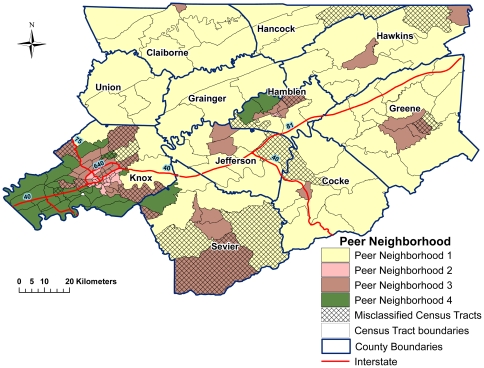
Identified peer neighborhoods (PN) in East Tennessee based on socioeconomic and demographic population characteristics using fuzzy K-means clustering algorithm.

**Figure 2:**
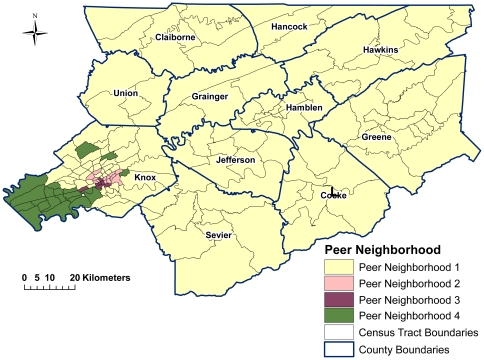
Identified peer neighborhoods (PN) in East Tennessee based on socioeconomic and demographic population characteristics using K-means clustering algorithm.

In sensitivity analyses, the solution that will provide the most insight into the data is one that has a higher FPU value and lower DPU value without being too close to a completely fuzzy solution (where FPU = 1 and DPU = 0). Thus, the results along with later validation revealed that the best clarity in neighborhood structure was achieved with the four PN solution at fuzzifier of 1.4 (Table 2). The optimum number of PNs could have been three, with very similar values for four PNs; however, indication from the fuzzy indices, a stronger classification rate, as well as, *a priori* knowledge of the study area, particularly the location of urban centers, indicated four PNs was the most sensible solution. The three PN solution tended to group small to medium sized urban centers (like those in Greene, Jefferson, and Sevier counties) with more rural neighborhoods, while the four PN solution separated them into different PNs (Figure 1). This is similar to, but not as exaggerated as, results from preliminary analyses of the data using hard clustering methods (K-means), where every neighborhood outside of Knox County was grouped into one PN (Figure 2) . Due to the known demographic diversity and socioeconomic variability of small to medium sized cities compared to rural neighborhoods in the study area, it was clear that those solutions (from standard k-means) were not providing good insight into the structure of neighborhood characteristics in the study area.

**Table 2:** Sensitivity Analysis of Fuzzy Cluster Analysis Results for Peer Neighborhoods Based on Socioeconomic and Demographic Population Characteristics.

Fuzzifier (m)	Three PNs		Four PNs		Five PNs		Six PNS	
	FPU*	DPU*	FPU	DPU	FPU	DPU	FPU	DPU
1.01	0.999	0.000	0.999	0.000	0.999	0.000	0.999	0.000
1.1	0.924	0.023	0.914	0.027	0.934	0.020	0.934	0.020
1.2	0.722	0.067	0.706	0.097	0.752	0.101	0.797	0.070
1.3	0.413	0.266	0.456	0.241	0.460	0.237	0.489	0.262
1.4	0.471	0.202	0.465	0.227	0.418	0.267	0.388	0.296
1.5	0.264	0.354	0.292	0.357	0.264	0.393	0.225	0.477
1.6	0.119	0.640	0.091	0.722	0.1352	0.650	0.110	0.691

DPU = Normalized average square error, values close to 1 are hard solutions; FPU = Dunn's normalized partition coefficient , values close to 1 are fuzzy solutions; PN = Peer Neighborhood.

*One wants to identify a solution that has a high FPU index and low DPU index without being too close to a completely fuzzy solution (where FPU = 1 and DPU = 0).

In the sensitivity analysis, one not only looks at the fuzzy indices, but also the patterns in membership belonging for neighborhoods in each PN as the fuzzifier changes. A summary of degrees of belonging for neighborhoods within each PN at different fuzzifiers is presented in Table 3. A stable neighborhood would have a primary (the PN to which it is classified) degree of belonging that is greater than 0.75. Fuzzy neighborhoods were described as having secondary and tertiary degrees of belonging greater than 0.25 to other PN(s) than the one in which it is classified. At m = 1.1, there are only 16 neighborhoods (9.6% of the total sample) with a secondary and tertiary degree of belonging of at least 0.25 or more. This indicates that these neighborhoods have a tendency to move elsewhere, i.e. have characteristics similar to another PN. At m = 1.3, 36.9% of the sample is showing this tendency, but more so the neighborhoods in PNs 3 and 4. At m = 1.4, the neighborhoods in PNs 3 and 4 are moving quickly toward diffused (or equal) degrees of belonging across all PNs, while PNs 1 and 2 are moving in that direction slowly. At m = 1.5, there is too much fuzziness since only a few neighborhoods in PN 1 (41.5%) have a strong degree of belonging to that PN. If there were no fuzziness in the clustering structure, these changes would not have occurred so quickly [35], [43]. Given that the desired solution should not be too fuzzy nor too hard, the suitable choices for the fuzzifier were m = 1.3 or 1.4. It would be expected that the fuzzy neighborhoods would form their own PN if the number of PNs was increased to 5 or 6 if the neighborhoods were uniquely different than the already established PNs, but this was not seen. Thus, the fuzzy observations actually lie in the space between the PNs, such that they are similar to more than one based on some characteristics. The fuzzifier m = 1.4 was chosen for the final solution because of the additional information it gave for some of the fuzzy neighborhoods, i.e. that they actually had similar characteristics to one or more other PNs, and because of the later strong validation with discriminant analysis and classification trees.

**Table 3:** Summary of Degrees of Belonging for Neighborhoods within Peer Neighborhoods as the Fuzzifier changes in Fuzzy Cluster Analysis.

PN	M = 1.1		M = 1.3		M = 1.4		M = 1.5	
	Stable^1^	Fuzzy^2^(%)	Stable	Fuzzy(%)	Stable	Fuzzy(%)	Stable	Fuzzy(%)
1	64	4 (5.9)	52	16 (23.9)	45	21 (31.8)	27	38 (58.5)
2	20	3 (13.0)	13	6 (31.6)	12	7 (36.8)	0	20 (100.0)
3	51	8 (15.0)	28	26 (48.1)	10	40 (80.0)	0	45 (100.0)
4	16	1(5.9)	13	14 (53.8)	7	25 (78.1)	0	37 (100)
**Total**	151	16 (9.6)	105	62 (36.9)	74	93 (55.7)	27	140 (83.8)

M = fuzzifier in fuzzy cluster analysis; PN = peer neighborhood.

1 The number of neighborhoods within the PN that are stable, i.e. have secondary or tertiary degrees of belonging to other PN(s) less than 0.25.

2 The number (%) of neighborhoods within the PN that are fuzzy, i.e. have secondary or tertiary degrees of belonging to other PN(s) greater than 0.25.

#### Characteristics of identified PNs

Peer neighborhood 1 was located primarily in rural, including the mountainous, areas (Figure 1) and was characterized by higher proportions of married people and homeowners, medium levels of income and housing value, but lower levels of education (Table 4). The most urbanized was PN 2, located in the downtown portions of cities with significantly lower median housing values, per capita income, education levels, proportion of homeowners, and proportion of married people compared to other PNs (Figure 1 & Table 4). This PN also had the highest proportions of single parent households, minorities, and younger populations. Peer neighborhood 3 was located in semi-urban areas and had the highest proportion of population ≥65 years, as well as the second highest levels of economic and higher education variables. Located in the suburban areas, PN 4 was the most affluent with significantly higher per capita income, housing value, employment, homeownership, and higher education (bachelor and graduate degrees) than other PNs (Figure 1 & Table 4).

**Table 4:** Summary Statistics of Socioeconomic and Demographic Population Characteristics of Peer Neighborhoods in East Tennessee.

	Peer Neighborhoods			
Variable	1	2	3	4
Living in urban areas (%)	11.4^C^	100.0^A^	87.5^B^	88.8^BA^
Below poverty (%)	17.1^B^	41.7^A^	15.1^B^	8.16^C^
Housing median value ($)	70741^BC^	36616^C^	83466^B^	128997^A^
Living in rural ares (%)	5.64^A^	0.00^B^	0.21^B^	0.26^B^
White (%)	97.4^A^	53.7^B^	91.4^A^	92.4^A^
Black (%)	1.11^B^	42.4^A^	5.13^B^	3.73^B^
Male (%)	49.6^A^	47.1^B^	48.1^AB^	49.0^A^
Population 40–59 yrs (%)	15.5^AB^	13.4^C^	14.8^BC^	16.7^C^
Population 50–59 yrs (%)	13.3^A^	8.69^C^	11.6^B^	13.0^AB^
Population 60–65 yrs (%)	5.38^A^	3.00^C^	4.43^B^	4.30^B^
65 yrs and over (%)	12.7^B^	11.4^B^	15.6^A^	12.6^B^
Single parent families (%)	6.89^BC^	17.3^A^	7.90^B^	5.03^C^
Average family size (#)	2.95^A^	2.99^A^	2.88^A^	2.93^A^
Married (%)	64.3^A^	34.1^C^	54.2^B^	62.0^A^
Employed (%)	55.3^B^	45.9^C^	58.3^B^	65.2^A^
Per capita income ($)	14795^B^	10735^C^	17654^B^	27859^A^
Homeowner (%)	81.8^A^	36.2^C^	63.1^B^	75.8^A^
Less than high school education (%)	36.5^A^	31.9^A^	25.7^B^	10.7^C^
High school graduate (%)	36.5^A^	29.2^B^	30.2^B^	18.7^C^
Some college (%)	18.8^B^	28.4^A^	26.6^A^	29.9^A^
Bachelor degree (%)	5.18^C^	5.98^C^	10.8^B^	22.8^A^
Graduate degree (%)	2.78^C^	3.32^CB^	5.29^B^	14.7^A^

A,B,C,D Mean separation based on Tukey (p<0.05) adjustment method. Means of the variable between peer neighborhoods that have the same letter are not significantly different.

#### Evaluation of misclassified neighborhoods

Both nearest neighbor DA and CART resulted in 86% correct classification of the four PNs (Table 5). This was by far the highest classification for any number of clusters (results not presented). The misclassified neighborhoods were often located along geographic borders of PNs (Figure 1). Thus, it was not surprising that these neighborhoods had degrees of belonging split between the PNs they bordered geographically. Additionally, the misclassified neighborhoods tended to be located just outside urban areas or areas that may have developing industry and/or transitioning population. For example, PN 1 had nine misclassified neighborhoods. The cross validation results in DA indicated that six of those nine were predicted to be in PN 3, while the other three were in PN 4. According to Hotelling's test, the six neighborhoods predicted for PN 3 had a significantly higher urban population while the three neighborhoods predicted for PN 4 had significantly higher housing values than the rest of the neighborhoods in PN 1. Similar results were found for misclassified neighborhoods in other PNs. The three misclassifications in PN 2 had significantly lower urban populations than the rest of the PNs and had equal degrees of belonging to PNs 2 and 3. PN 3 had the most misclassifications with 10 neighborhoods predicted to be either in PN 1 (if they had a significantly lower proportion of urban population) or in PN 4 (if they had significantly higher median housing values and lower proportions of the population living below poverty). The least number of misclassified neighborhoods occurred in PN 4 where two neighborhoods were predicted to belong in PN 3. However, no differences in socioeconomic and demographic characteristics from Hotelling's test were found.

**Table 5:** Nearest Neighbor Discriminant Analysis Results of Classification of East Tennessee Peer Neighborhoods Based on Socioeconomic & Demographic Characteristics.

	Actual Peer Neighborhood				
Predicted	1	2	3	4	Total
**1**	57	0	3	0	60
**2**	0	16	0	0	16
**3**	6	3	40	2	51
**4**	3	0	7	30	40
**Total**	66	19	50	32	167

#### Variables important for classifying PNs

**Figure 3:**
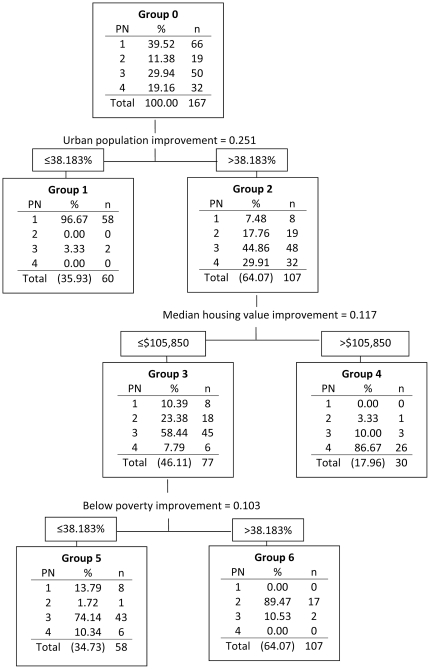
Cluster and regression tree (CART) results for peer neighborhoods (PNs) in East Tennessee.

CART results show that the first split was on percent urban population ≤38.183% leading to 97% correct classification in PN 1 (Figure 3). The second split occurred with percent urban population >38.183 and housing value >$105,850. This resulted in 87% correct classification in PN 4. The third split occurred when percent urban population was greater than 38.183% and housing value ≤$105,850. This produced two groups with percent below poverty level ≤27.276% yielding a 74% correct classification in PN 3. When the percentage below poverty level was >27.276%, 89% of the neighborhoods were correctly classified in PN 2.

Given that the CART and DA yielded similar classification results, the uniqueness of the four identified PNs was supported. The percent of population living in urban areas, the median housing value, and the percent of population living below poverty in a neighborhood were the most important variables in determining correct classification of neighborhoods.

### Disparities in stroke and MI mortality between PNs

**Figure 4:**
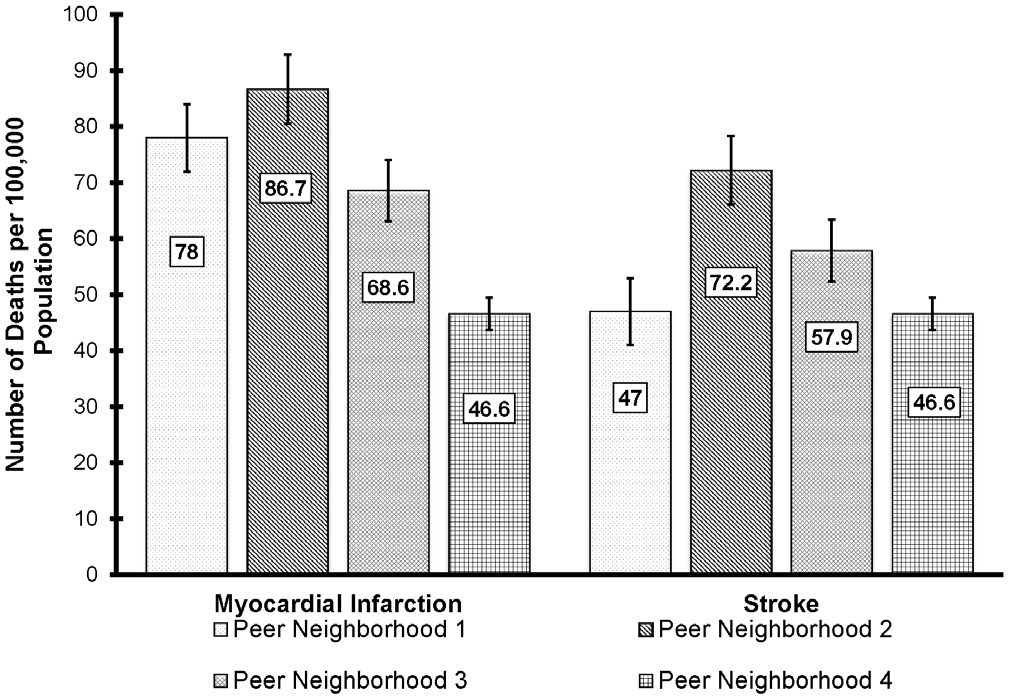
Annual age-adjusted stroke and myocardial infarction mortality risks for peer neighborhoods in East Tennessee.

Peer neighborhood 4, the most affluent PN and located in the suburbs, had significantly lower (p = 0.01) risks for stroke and MI mortality than all other PNs (Figure 4). Conversely, the most urban and least affluent neighborhood, PN 2, tended to have higher risks of stroke and MI mortality, although these were not significantly (p = 0.6) different from the risks for both PN 1 and PN 3. Only the MI mortality risk for PN 2 was greater than the state risk of 85.5/100,000, while the risk for PN 4 was the only one below the US risk (58.9/100,000). The stroke mortality risks in PNs 2 and 3 exceeded both the state (67.5/100,000) and US (53.5/100,000) risks.

## Discussion

To our knowledge, this is the first study to investigate the clustering of population characteristics that are associated with stroke or MI at the neighborhood level. Based on knowledge of the study area, the four PNs identified are a unique and sensible classification of neighborhoods based on socioeconomic, demographic, geographic characteristics for East Tennessee. The geographic distribution of identified PNs revealed that the most affluent neighborhoods are located in suburban areas, while the least affluent neighborhoods were located in the downtown areas. These findings are consistent with those from other studies that have investigated neighborhood level socioeconomic and demographic determinants of health [21], [50].

Several studies have considered socioeconomic or demographic characteristics of populations in relation to stroke or MI, but historically most of these analyses have been done at state [1], [3], [4], [51], [52] or county [53], [54] geographic levels. Recent studies indicate that finer geographic units are needed to increase the clarity of distributions of determinants of health [7], [18] and to better guide local health planning and targeted health programs. To address this issue, the current study was performed at the census tract level, which have been found to be good proxies of natural neighborhoods [21], [25]. Additionally, census tract level socioeconomic and demographic data are available to all states in the US through the US Census Bureau, as well as for populations in other countries like Canada (census tracts) [55] and the United Kingdom (postcode sectors) that approximately correspond to US census tracts [19], [56]. Given the lack of socioeconomic information provided in US vital records, population studies must rely on census data in order to investigate population characteristics at a neighborhood level. Comprehensive data at the census tract level is also limited by the decennial nature of the US census, such that the data may be outdated or not accurately reflect neighborhood composition due to population growth and migration. To address this issue, it has been recommended that only data from the closest census falling within five years of the study period should be used [57]. Thus, the 2000 census data were best suited to match the disease data (1999–2007) for this study. Furthermore, the 2010 census data were not available at the time of this study's analyses. Since census tract level was the best available data for the current study, robust multivariate methods were utilized to be able to include many socioeconomic and demographic variables in order to reduce bias and get the most comprehensive insight into neighborhood characteristics of the study area. As this was a population health planning approach and the goal was to better understand neighborhood effects, individual level risk factors (like genetics, co-morbidities, medical history, or modifiable behaviors) that may affect stroke or MI patterns [27], [58] were not included in the analyses. Although census data are useful and are currently the best available data for these types of analyses to address these types of research and health planning questions, they are not without limitations. Some of the limitations associated with census data include both sampling (e.g. missing street address) and non-sampling errors (e.g. phrasing of questions which may influence the response) and hence the data obtained [59], [60].

The association of socioeconomic and demographic characteristics with survival after MI at the neighborhood level has been described by other studies using census tracts as the geographic unit of analysis [11], [61]. However, these studies included only one or a few demographic factors and measures of socioeconomic status. Other studies have found that neighborhood SES is important in determining risk using composite socioeconomic and demographic measures [8], [62]. Evidence from recent research indicates that many socioeconomic and demographic characteristics are not interchangeable, and so the use of one measure or a composite measure ignores the complex relationships between the factors [11], [18]. The results from the robust PCA in this study also indicated that, despite high correlations between variables, additional information existed that would be lost if some variable(s) were removed. For instance, while many of the variables that heavily loaded on component 1 were highly correlated, their loadings differed across the other components. Thus, the variables were explaining different pieces of information or variation across those components. These complex interrelationships among socioeconomic and demographic factors imply that as many risk factors as realistically possible are needed for the most holistic analysis.

When using a high number of risk factors to classify neighborhoods into similar groups, issues with interdependencies among variables, different variable scales, and outliers are likely to arise. A major strength of this study was the use of robust PCA to account for these issues and reduce their bias on cluster analysis [35]. Furthermore, the fuzzy cluster strategy was utilized to allow neighborhoods to have associations with more than one PN, giving insight into the structure of the data when groups may not be mutually exclusive [43]. The drastic difference in results (Figures 1 and 2) revealed that the fuzzy clustering approach provided more insight into the true structure of the neighborhoods, while the traditional k-means approach seemed to be more influenced by outliers in Knox county, masking the characteristics of some neighborhoods in other counties. The complex interrelationships between the risk factors and the multi-factorial nature of causation of stroke and MI indicate that some overlap between groups could be expected. These areas of overlap are particularly important when considering neighborhood health needs since the identified unique population profiles are valuable in the development of population health programs. Information on the tendency of a neighborhood to move toward another PN from the sensitivity analysis of the fuzzy method is very useful when developing population health programs since every neighborhood is important. This allows health initiatives to be targeted at the neighborhood level based on the population characteristics and health needs, instead of a larger area that has more diverse characteristics. The implication of this is that, within an administrative unit (such as a county), health professionals are able to use a needs-based approach to planning and service provision, based on unique neighborhood profiles and health needs, instead of using a “one-size-fits-all” strategy. Thus, within an administrative unit, different programs can be designed to meet the distinct needs of the different neighborhood types based on their unique profiles.

In order to get the most comprehensive idea of the structure of the neighborhoods and to fully understand the uniqueness of those misclassified observations where overlap between PNs could be expected, it was important to explore the cluster solution using several validation methods. The majority of misclassified neighborhoods were found in PNs 3 and 1. This was expected given that these PNs had levels of socioeconomic and demographic variables somewhere in between the distinct high and low extremes of PNs 4 and 2, respectively (Table 2). The fuzzy analysis allows the overlap of the misclassified neighborhoods with fuzzy degrees of belonging across another PN to be highlighted. This implies that it may be necessary to consider some neighborhoods in more than one PN in the population health planning of those different areas. For example, when designing a targeted health program for improving heart attack mortality risk for PN 3, one would also want to consider those neighborhoods classified as PN 2 but had high degrees of belonging (i.e. similar characteristic) to PN 3. Though these neighborhoods were classified in PN 2 because of their urban locations, their demographic and socioeconomic characteristics were more consistent with PN 3. Thus we would expect health needs for these neighborhoods to be similar to PN 3. Practically, heart health education campaigns, such as diet and exercise recommendations, geared toward less diverse and higher income populations like PN 3, might be additionally presented to those neighborhoods in PN 2 that were similar to PN 3. Therefore, in addition to statistical analyses, visual evaluation of the grouping of neighborhood characteristics into PNs and the prior knowledge of relationships between the variables and health outcome of interest are important in recognizing patterns that are useful in aiding resource allocation and service provision.

Several studies have found that risks of stroke and MI are inversely related to socioeconomic factors like education and income and positively associated with demographic factors like proportion of males, blacks, and population over 65 [3], [51], [52]. In this study, these characteristics were clustered in neighborhoods located in the most urbanized downtown areas. Similar results have been reported by a Canadian study [21]. In addition to urbanicity, the current study also found that median housing value and the proportion of the population living in poverty were the key factors in classifying PNs. While urban populations have not been directly reported to have increased stroke and MI risk, they tend to have socioeconomic and demographic factors consistent with increased risk, i.e. tend to be the less affluent segments of the population.

Indeed, this study found that a significant disparity exists in both stroke and MI mortality between less affluent, urbanized neighborhoods and more affluent, suburban neighborhoods. This is very concerning since recent reports indicate that the disparity in cardiovascular death risks is widening between lower and higher socioeconomic status groups [7]. This study provides information on the unique socioeconomic and demographic profiles of neighborhoods that can aid in understanding disparities in health outcomes by identifying the unique challenges and health needs between neighborhoods. For instance, although PNs 1 and 3 seem to have similar socioeconomic characteristics, close evaluation reveals that these PNs greatly differ. PN 3 has a significantly more urban, older, and educated population than PN 1. If only socioeconomic characteristics are considered, these populations would incorrectly be considered similar. From a health planning perspective, it is clear that older populations, like PN 3, would have different health needs than other segments of the population. Additionally, PNs 1 and 4 have similar stroke risks (47 and 46.6 annual deaths per 100,000 population, respectively). Both PNs have higher levels of income; however, PN 4 is a somewhat younger, more urban and more ethnically diverse than PN 1. Thus, different characteristics at the neighborhood level must be considered in targeting health education and outreach activities in order to improve outcomes and reduce disparities.

The neighborhood focused approach of this study is applicable to health planning in areas other than East Tennessee. The generalizability is not specifically in the study findings, but in the application of the methodology to provide insight into the unique population characteristics and potential health needs of other communities based on empirical evidence. The findings of this study serve as examples of the type of information that can be obtained from this approach and its usefulness from a population health planning perspective. It would be expected that a different number of PNs with different sets of unique profiles would be identified using this methodology in different populations. However, the health outcome improvement programs and health disparity reduction strategies could then be specifically tailored to the results and specific needs of neighborhoods of interest.

In conclusion, the robust and fuzzy multivariate techniques utilized in this study to classify neighborhoods based on socioeconomic or demographic characteristics identified four unique population profiles in the study area. Stroke and MI mortality risk differed between the identified PNs. The PNs with highest mortality risk also had the highest levels of socioeconomic variables known or suspected to be associated with higher risk of stroke or MI and were located in the urbanized downtown areas. The lowest mortality risk was associated with the most affluent PN. These findings provide population health planners a unique opportunity to better understand and effectively plan for the unique neighborhood health needs. Thus, implementation of these methodologies and careful integration of the findings in health planning activities will be useful in guiding health resource allocation, service provision, and policy decisions at the local level. Moreover, this information is important for addressing neighborhood health disparities not only in the East Tennessee Appalachian Region, but also for other health planning regions throughout the US and other countries given the availability of socioeconomic and demographic data.
